# Kinetics of Circulating Progenitor Cells and Chemotactic Factors in Full-Term Neonates with Encephalopathy: Indications of Participation in the Endogenous Regenerative Process

**DOI:** 10.3390/biom15030427

**Published:** 2025-03-17

**Authors:** Nikolaos Efstathiou, Georgios Koliakos, Katerina Kantziou, Georgios Kyriazis, Aristeidis Slavakis, Vasiliki Drossou, Vasiliki Soubasi

**Affiliations:** 1 1st Neonatal Clinic and NICU, Hippokration General Hospital, Aristotle University of Thessaloniki, 54642 Thessaloniki, Greece; 2 Biochemistry Department, Medical School, Aristotle University of Thessaloniki, 54124 Thessaloniki, Greece; 3 Immunology Department, Pulmonary Clinic, Papanikolaou General Hospital, Aristotle University of Thessaloniki, Exohi, 57010 Thessaloniki, Greece; 4 Biochemistry Department, Hippokration General Hospital, 54642 Thessaloniki, Greece

**Keywords:** progenitor cells, neonate, encephalopathy, full-term, brain, circulating, haematopoietic stem cells, very small embryonic-like stem cells, endothelial progenitor cells

## Abstract

Preclinical studies have shown that progenitor cells (PCs) are mobilized toward injured tissues to ameliorate damage and contribute to regeneration. The exogenous therapeutic administration of PCs in children affected by neonatal encephalopathy (NE) is a promising, yet underreported, topic. In this prospective study, we investigated whether endogenous circulating progenitor cells (CPCs) are involved in intrinsic regeneration mechanisms following neonatal brain injury. Thirteen full-term infants with moderate/severe NE, eleven with perinatal stress, and twelve controls were enrolled. Blood samples were collected on days 1, 3, 9, 18, and 45, as well as at 8 and 24 months of life, and were analyzed with a focus on Endothelial Progenitor Cells, Haematopoietic Stem Cells, and Very Small Embryonic-Like Stem Cells, in addition to chemotactic factors (erythropoietin, IGF-1, and SDF-1). Correlations between CPCs, chemotactic factors, and brain injury were assessed using serum levels of brain injury biomarkers (S100B and neuron-specific enolase), brain MRIs, and Bayley III developmental scores. Increased brain injury biomarkers were followed by the upregulation of SDF-1 receptor and erythropoietin and, finally, by elevated CPCs. These findings suggest a potential endogenous regenerative effort, primarily observed in the moderate encephalopathy group, but this is suppressed in cases of severe brain injury. Mimicking and enhancing endogenous regeneration pathways in cases of failure—regarding cell type and timeframe—could provide a novel therapeutic model.

## 1. Introduction

Perinatal asphyxia and subsequent neonatal encephalopathy constitute a frequent cause of neonatal death and long-term neurodevelopmental deficits, such as cerebral palsy, epilepsy, learning difficulties, and mental retardation [1]. Current therapeutic approaches are primarily supportive, with the exception of therapeutic hypothermia, which is mostly beneficial in a subgroup of neonates (i.e., those with a moderate degree of encephalopathy when applied within the first 6 h of life, according to the current inclusion criteria) [1,2]. Rapid developments in regenerative medicine and increasing knowledge in neurogenesis are increasingly illuminating the possibilities for repairing neuronal damage following hypoxic-ischemic encephalopathy. Specifically, research interest is focused on the role of progenitor/stem cells and their possible exogenous administration after neonatal brain injury. Progenitor cells are multipotent cells of primitive origin that have the ability to self-renew and differentiate into multiple different cell lines (plasticity) [3,4]. During the embryonic period, they are involved in organogenesis, while during extra-uterine life, they are capable of cell renewal after apoptosis and tissue repair. They home in almost any tissue, but mainly in the bone marrow, and mobilize rapidly into the blood stream (circulating progenitor cells, i.e., CPCs) in a chemotactic way towards an injured tissue that releases chemoattractants [3,5,6,7,8,9,10,11]. Progenitor cells are mobilized locally [12] after brain damage but also from the opposite brain hemisphere [13] and from the periphery [4,14,15,16], especially considering that the blood–brain barrier is more permeable in cases of brain injury. Preclinical studies have shown that these cells can ameliorate tissue damage due to their immunomodulatory, trophic, neoangiogenetic, and neurogenetic properties [17,18,19]. A plethora of studies on animal models have shown favorable results [12,13,14], where the first trials in human neonates have confirmed the safety of the method and have shown some indications for positive results [20,21,22,23,24]. Favorable results are mainly short-term or refer to the tissue level, while long-term clinical improvement is indeed controversial [9,20,22,23,25,26,27,28,29]. In this early stage, a multitude of centers around the world have started exogenous administration, hoping to improve brain injury in infants and children, without any well-reported results. Additionally, issues of optimum cell type, dosage, route, and timeframe of administration still need to be addressed.

On the other hand, it is known that those progenitor cell lines do exist in the body of an infant, circulating in the peripheral blood and homing mainly in the bone marrow, arising very critical issues that have to be investigated: are these cells mobilized after brain injury? Do they take part in an endogenous effort of regeneration? If they have no role after brain damage, how is it possible that enhancing their number after exogenous administration would be beneficial? Evolution throughout centuries has chosen certain biochemical tracts and cells as the optimum factors for tissue regeneration, similar to conducting thousands of trials and picking the most effective one every time. Many of the most successful current therapeutics are based on mimicking the endogenous response [30,31,32]. It is quite clear that if endogenous progenitor cells do not have any role after brain injury, they would not have any role after exogenous administration either, at least without any concomitantly complex chemoattractant or genetic intervention.

Aiming to fill the gap between preclinical studies and intervention trials in infants, we aimed to investigate the possible role of endogenously circulating progenitor cells in neonatal encephalopathy. Specifically, this study aimed (a) to illustrate and analyze the related pathophysiology regarding the sequence of events (timeframe and severity of brain injury), possible increase in neurotrophic/chemoattractant factors, and possible increase in CPCs and (b) to investigate which clinical parameters influence the above kinetics and, most importantly, determine the correlation with the short-term and long-term neurodevelopmental outcome.

In this study we focused on Haematopoietic Stem Cells (HSCs), Very Small Embryonic-Like Stem Cells (VSELs), and Endothelial Progenitor Cells (EPCs), which are some of the most promising progenitor cell lines that have been studied.

HSCs are primarily found in the bone marrow and take part in haematopoiesis [33], but they are also involved in tissue regeneration after injury [34], promoting the chemotaxis, survival, and development of both local cells and other progenitor cell types [35]. They also play a role in angiogenesis, contributing EPCs [36,37]. Under normal homeostasis, HSCs are found in low numbers in peripheral blood. However, in response to tissue damage, they rapidly mobilize from the bone marrow to the systemic circulation through the SDF-1/CXCR4 axis [38,39,40,41]. Beneficial HSC mobilization has been shown in preclinical [42] and clinical studies on adults [43,44,45] after ischemic stroke. Generally, it seems that HSCs are increased in the acute phase of tissue injury (probably contributing to tissue regeneration), while in chronic diseases, their levels are low (partly explaining impaired rehabilitation) [46,47,48]. An inconsistency in HSC characterization among studies is a great obstacle in reaching common conclusions. Overall, HSCs play a key role in homeostasis and tissue repair, making them a promising tool in regenerative medicine.

VSELs are pluripotent progenitor cells of non-haemopoietic origin, found mainly in the bone marrow and other tissues such as cord blood, brain, kidneys, pancreas, liver, and muscles. They are involved in organogenesis in utero and in tissue regeneration [49] in the extra-uterine life, mobilized rapidly in the blood through the SDF-1/CXCR4 and Hepatocyte Growth Factor/c-met axis [49,50]. Preclinical [50] and clinical studies on adults [51,52] have shown hopeful involvement after a stroke. Observations in premature neonates are still inconclusive [53,54]. Overall, VSELs hold significant potential for regenerative medicine, especially for neural tissue repair, but further research is needed to fully understand their capabilities and applications.

EPCs, first identified in 1997, are cells found in peripheral blood, bone marrow, and umbilical cord blood that contribute to vascular repair and neovascularization, playing a role in tissue recovery after injury [55,56,57,58]. Their mobilization is driven by cytokines like erythropoietin and vascular endothelial growth factor (VEGF) [59,60]. EPCs are important indicators of vascular regeneration, with their numbers found to be decreased in chronic diseases like coronary artery disease, diabetes, and hypertension, and are often linked to poorer outcomes as well [61,62,63]. Conversely, in acute conditions like myocardial infarction, higher EPC levels correlate with better recovery [64,65]. Circulating EPCs are very challenging to identify due to their heterogeneous stages of differentiation and overlapping surface markers with mature endothelial cells. Recent research suggests that EPCs should be identified as CD34^+^, VEGFR2^+^, and CD45^−^ (or CD45^dim/–^) [56,60,66,67,68,69], while CD133 is not a specific marker; rather, it is mostly a marker for early progenitors [60,70]. Researchers continue to debate the proper identification of EPCs due to the complexity and variation in surface marker expression.

Chemotactic factors mostly related to the abovementioned CPCs are erythropoietin (EPO), insulin-like growth factor-1 (IGF-1), and stromal cell-derived factor-1 (SDF-1).

EPO is a glycoprotein with chemotactic, pleiotropic growth, and neuroprotective properties [71,72]. While its primary function is in erythropoiesis, growing evidence supports its potential role in influencing a variety of biological processes, including the regulation of inflammation and immune response, angiogenesis and neurogenesis during both embryogenesis and in vitro conditions [73]. The low partial oxygen pressure in tissues induces the synthesis of the hypoxia-inducible factor-1a (HIF-1a), which subsequently triggers the production of erythropoietin. Evidence for its neuroprotective effects has also been shown in studies on neonates [74,75,76,77,78]. EPO appeared to be produced endogenously within the brain following injury and was positively associated with related outcomes [79]. The neuroprotective effects of the exogenous peripheral administration of EPO are likely due to indirect actions, possibly via the mobilization of progenitor cells [79]. EPO has long been known to influence the mobilization of CD34^+^ CD45^+^ progenitor cells and EPCs into peripheral blood in a dose-dependent manner [80], although this finding is not consistent across studies [60,81]. EPO is certainly one of the most promising therapeutic factors in brain injury.

IGF-1 plays a vital role in embryonic growth, brain development, and the differentiation and protection of pre-oligodendrocytes [82,83]. Hypoxia induces the HIF-1a factor, which, in turn, promotes an increase in the levels of IGF-1 [73], which has anti-apoptotic effect [84] and promotes the survival of neural cells after neonatal encephalopathy [1,83,85]. Several preclinical models verify its neuroprotection actions [86,87]. IGF-1 levels rise during embryonic life [88], and its levels may be linked to physical growth and brain maturation and protection [89,90]. IGF-1’s neuroprotective potential makes it a promising therapeutic target for improving brain development after injury.

SDF-1 is a chemotactic factor that binds to the CXCR4 receptor (CD184) on progenitor cells, including neural stem cells and CPCs [91]. In uterus, it plays a critical role in brain development, guiding neuroblast migration and angiogenesis [91]. In post-embryonic life, SDF-1 is involved in anchoring progenitor cells in the bone marrow and directing them to ischemic tissues, resulting in increased angiogenesis [92,93]. After ischemic injury, HIF-1a increases SDF-1 production in the endothelial cells near the damaged site, which, in turn, increases the expression of the SDF-1 receptor on CPCs, directing them to the injured site [38,91]. Clinical studies on adults have shown increased SDF-1 levels in ischemic tissues and in blood, which correlates with elevated progenitor cell numbers [52,94,95]. It seems that the SDF-1/CXCR4 axis plays a crucial role in the migration, differentiation, survival, anchorage, and quiescence of progenitor cells in the bone marrow.

Aiming to correlate cells’ and chemoattractants’ kinetics with the brain injury event, serum brain biomarkers, brain MRI imaging, and neurodevelopmental tests were used to delineate the devastating event as accurately as possible. Among brain biomarkers, S100B and Neuron-Specific Enolase (NSE) have proven to be more hopeful in preclinical and clinical studies [96]. Specifically, S100B, a calcium-binding protein produced in astrocytes and other glial cell types, is released after cell death, with its levels increasing in cerebrospinal fluid (CSF), blood, and urine. Its small molecular weight allows it to pass through the blood–brain barrier (BBB), especially in cases of brain injury [96]. NSE is a glucose metabolism-related isoenzyme that is produced mainly in neurons and neuroendocrine cells. After brain injury and impairment of BBB, the concentration of both S100B and NSE are elevated in peripheral blood [97].

**Figure 1:**
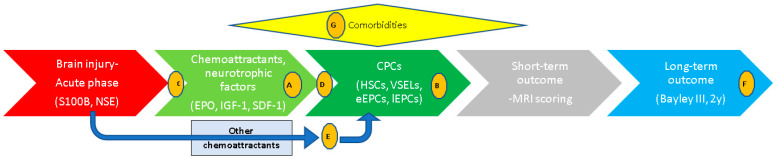
Schematic representation of events related to neonatal encephalopathy and hypotheses investigated in the current study. Letters in circles indicate the hypothesis studied. Hypothesis A–G: (A) kinetics of neurotrophic and chemotactic factors (EPO, IGF-1, and SDF-1). (B) Kinetics of CPCs (HSCs, VSELs, early EPCs (eEPCs), late EPCs (lEPCs)). (C) Do brain injury markers correlate with the chemotactic factor measured in this study (EPO, IGF-1, and SDF-1)? (D) Do these chemotactic factors correlate with CPC kinetics? (E) Do brain injury markers correlate with CPC kinetics via other (not studied here) chemoattractants? (F) Is long-term neurodevelopmental outcome correlated with the parameters studied? (G) Are there indications that comorbidities influence those parameters?

A single-center pilot translational study was conducted in a neonatal intensive care unit of a tertiary hospital to illustrate the related pathophysiology and endogenous regeneration biochemical pathways directly in human full-term neonates with encephalopathy and aimed to confirm if preclinical observations are valid in real clinical situations. Specifically, we investigated if signals for neuroprotection (chemoattractants) are sent in neonatal encephalopathy (NE) (hypothesis A, Figure 1) and if recipients of these signals (i.e., CPCs) are mobilized (hypothesis B), identifying malfunctioning endogenous repair mechanisms (in terms of cell type and timeframe) and opening new horizons for the prevention of long-term neurological disabilities. A schematic representation of the parameters and hypotheses tested in the current study are represented in Figure 1. A constant pattern of a successful endogenous regeneration effort in neonates with moderate encephalopathy is highlighted in this study, where these repair mechanisms seemed to fail in severe brain injury.

## 2. Materials and Methods

This is a prospective cohort study on full-term newborns who underwent a perinatal sentinel event followed by the clinical spectrum of neonatal encephalopathy. A perinatal sentinel event was defined by the presence of three or more of the following: perinatal hypoxic event, 5′ min Apgar score lower than 5, arterial pH lower than 7 and base deficit higher than 12 in the first blood sampling, need for resuscitation, need for oxygen therapy, sudden and sustained fetal bradycardia, absence of fetal heart rate variability, and/or persistent late or variable decelerations. Neonates with brain injury were divided into the three distinct grades of encephalopathy (mild, moderate, and severe), as Sarnat and Sarnat have described [98]: mild encephalopathy, which lasts less than 24 h and is characterized by hyperalertness, uninhibited Moro and stretch reflexes, sympathetic effects, and a normal electroencephalogram; moderate encephalopathy, which lasts for 2–14 days and is characterized by obtundation, hypotonia, strong distal flexion, multifocal seizures, and characteristic electroencephalographic periodic patterns; and severe encephalopathy, which lasts days or weeks, where infants are stuporous and flaccid, with brain stem and autonomic functions being suppressed. Newborns who were hospitalized at our tertiary neonatal intensive care unit within the first 24 h of life were enrolled and divided retrospectively (on the 3rd day of life) into study groups according to their neurological examination and the related prognosis: neonates who were considered as high-risk for adverse outcome (neonates with moderate or severe neonatal encephalopathy) were defined as the patient group [98], while neonates with a low risk for a significant adverse neurological outcome and a favorable prognosis were grouped separately (mild encephalopathy group). Additionally, a cohort of healthy full-term newborns, selected randomly (in specific time points, given the parents’ approval) from the obstetric department, served as controls. All infants were prospectively followed up until after the second year of life. The exclusion criteria were considered conditions that could influence the parameters studied, i.e., metabolic disorders, intrauterine growth retardation, congenital coagulopathies, major congenital abnormalities, and twins with death or major abnormalities in their co-twins [99,100,101,102]. Hemolyzed samples were also excluded. Demographic and neonatal characteristics, as well as biochemical parameters, were recorded. Sepsis was defined as a positive blood culture. A subanalysis of the data from the patient group into discrete subgroups of moderate and severe encephalopathy was used for the optimal visualization of kinetics and to help understand the underlying pathophysiology. This study was approved by the Scientific Committee of Hippokration Hospital and the Ethical Committee of the Faculty of Medicine, Aristotle University of Thessaloniki. Written consent was obtained from the parents of all the newborns.

Peripheral blood samples were obtained on days (d) 1 (specifically between the 12th and 24th hours of life), 3, 9, 18, and 45 of life as well as in the 8th month and after the 24th month of life (between 24 and 28 months). Peripheral blood was collected into tubes containing the anticoagulant ethylenediaminetetraacetic acid (EDTA). A volume of 100 µL of blood was incubated for 15 min in 15 × 75 mm tubes after the addition of 10 µL of each of the following four monoclonal antibodies: anti-CD184 (CXCR4)-PE, anti-CD34-FITC, anti-CD45-PECy5 (BD Biosciences, San Jose, CA, USA), and anti-CD133-APC (Miltenyi Biotec, Bergisch Gladbach, Germany). The incubation was performed at room temperature and in the dark. Simultaneously, the blood was incubated with the corresponding four isotype controls: mouse IgG2a-PE, mouse IgG1-FITC, mouse IgG1-PECy5, and mouse IgG1-APC (EXBIO, Prague, Czech Republic), which were used as negative controls (to account for nonspecific fluorescence). For the comparison of the diameter of biological microparticles and cells from the populations under study, beads with diameters of 0.5 µm, 0.9 µm, and 3 µm (Megamix beads, Biocytex, Marseille, France) were used. After incubation, red blood cells were lysed by adding a lysing solution (BD PharmLyse Lysing Solution, BD Biosciences, San Jose, CA, USA), incubated for 15 min in the dark at room temperature, and washed with PBS (Phosphate-Buffered Saline). Measurements were performed on a four-color flow cytometer, FACSCalibur (BD Biosciences, Mountain View, CA, USA), using the BD FACSDiva Software (version 6, BD Biosciences, Mountain View, CA, USA). All measurements were performed in duplicate, and the averages were calculated. The results are presented as dot plots, with each point representing a cell and the logarithms of the fluorescence intensities of the fluorochromes plotted on the x- and y-axes. CPCs of interest were populations enriched in HSCs (CD34^+^/CD184^+^/CD45^+^), VSELs (CD34^+^/CD184^+^/CD45^−^), eEPCs (CD34^+^/CD45^dim/−^/CD184^+^/CD133^+^), and lECPs (CD34^+^/CD45^dim/−^/CD184^+^/CD133^−^). For each sample, 100.000 events were acquired. The levels of each cell population are expressed as a percentage of the total events. A detailed description of flow cytometry methodology and gating strategy has been described earlier [4].

From the same blood samples, serum and plasma were collected and stored at −80 °C until measurement of brain injury biomarkers and chemoattractants. S100B and NSE were assessed using an immunochemiluminometric assay (ICMA) (Liaison, DiaSorin-SpA, Saluggia, Italy), and the blood samples were processed according to the manufacturer’s instructions, as described earlier [97]. EPO and IGF-1 levels were measured in serum samples using an immunochemiluminometric assay (Immulite 2000XPi, Siemens, Llanberis, UK), and SDF-1 levels were assessed in plasma samples using ELISA (Quantikine ELISA, R & D Systems, Minneapolis, MN, USA).

Brain MRI (1.5 T) was performed in neonates with moderate or severe NE during their second week of life. Axial, coronal, and sagittal T1/T2-weighted images were evaluated. MRI scores of basal ganglia injury and watershed pattern of injury were calculated (as Barkovich et al. have proposed [103,104]) by a pediatric neuroradiologist who was blinded to all clinical parameters and outcomes. Brain MRI is not performed in healthy neonates and in neonates with mild perinatal stress in our clinical practice. Normal MRI scores [103,104] were used for comparison with MRI scores of neonates with moderate/severe encephalopathy.

Neurodevelopmental assessment with the Baley III test was performed after the second year of life by an experienced investigator who was blinded to clinical parameters. The Baley III test examines cognitive, language (receptive and expressive), and motor (fine and gross) domains [105].

Statistical analysis was performed using the SPSS statistical package (SPSS statistics, IBM Corporation, version 20, Chicago, IL, USA). Sample size analysis was used in order to indicate the lowest sample size required to detect statistical significance. Alpha was set to 0.05, and 80% power was accepted. Calculating for an additional 10% drop-out rate, the estimated sample size was defined as 11 neonates per arm. The data are presented as mean (±SD) or median (range) depending on the distribution, and a two-tailed *t*-test or the Mann–Whitney U-test was used for quantitative parameters, respectively. Likewise, correlations were evaluated using Pearson’s correlation or Spearman’s rank correlation, as appropriate. Pearson Chi-Square or Fisher’s exact tests were used for qualitative variables. Receiver operating characteristic (ROC) curves were assessed using the areas under the curves as indicated. Cutoff points were selected to optimize the overall predictive ability. A *p*-value of less than 0.05 was considered as statistically significant. As this is a pilot study, when rational causalities exist, borderline *p*-values (0.05 > *p* > 0.08) were also reported to indicate trends.

## 3. Results

### 3.1. Neonatal Characteristics

Forty full-term neonates were enrolled in this study (88.8% parents’ consent rate). Two newborns who fulfilled the exclusion criteria (one with congenital heart disease and one with possible metabolic syndrome) and two who were lost to follow-up were excluded from this study. Twelve healthy full-term neonates served as controls (group 1). Eleven newborns sustained mild perinatal stress/asphyxia and showed only minor neurological signs during the first 24 h of life, after which they had normal clinical appearance (mild encephalopathy, group 2). Finally, 13 neonates sustained moderate (n = 5) or severe (n = 8) neonatal encephalopathy after perinatal asphyxia and were considered as high-risk for an adverse outcome (patients, group 3). The neonatal characteristics are presented in Table 1. Two neonates with moderate and two with severe encephalopathy did not undergo therapeutic hypothermia, as defined by clinical protocols, because they did not meet the criteria by the 6th hour of life or due to late transportation to the tertiary hospital, where this study took place.

### 3.2. Erythropoietin

#### 3.2.1. Kinetics

**Figure 2:**
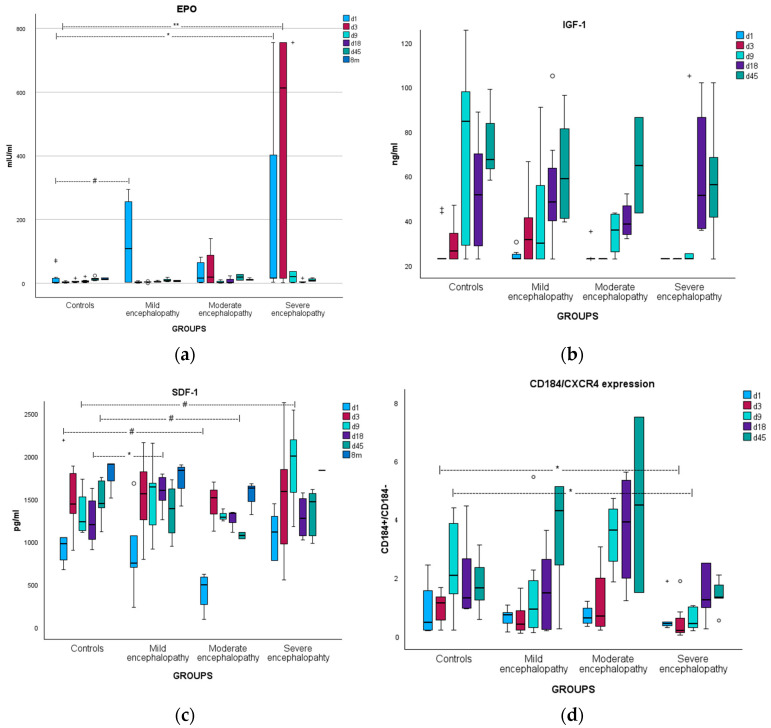
Illustration of kinetics in neonates with different levels of encephalopathy and controls of (**a**) EPO levels; (**b**) IGF-1 levels; (**c**) SDF-1 levels; and (**d**) CD184 receptor expression on the surface of CPCs. * *p* < 0.05; ** *p* < 0.005; and # trend (0.05 < *p* < 0.08).

EPO levels were significantly increased in patients on days 1 and 3 of life compared to controls. A subanalysis showed that this increase was mainly observed in the subgroup of severe encephalopathy from day 1 to day 9 of life. A marginal increase was also found in neonates with mild encephalopathy on day 1 (Table 2 and Figure 2a).

#### 3.2.2. Correlations

EPO levels were correlated with CPCs only in the group of patients. Specifically, EPO levels on days 3 and 9 were correlated with VSELs on day 45 (rho = 0.703/*p* = 0.078 and rho = 0.857/*p* < 0.05, respectively). Additionally, EPO levels (d1) were correlated with eEPCs (d18) (rho = 0.697/*p* < 0.05), whereas EPO levels on day 3 were borderline correlated with eEPCs of day 45 (rho = 0.685/*p* = 0.085). Finally, EPO levels (d9) were marginally correlated with lEPCs (d45) (rho = 0.715/*p* = 0.071). Low pH after birth was correlated with increased levels of EPO on day 3 (rho = −0.62/*p* < 0.01).

### 3.3. Insulin-like Growth Factor-1

#### 3.3.1. Kinetics

Patients had marginally lower IGF-1 levels on days 3 and 9 of life (Table 3). There was no difference between the subgroups of moderate and severe encephalopathy. IGF-1 levels gradually increased during the first 45 days of life in all groups (Figure 2b).

#### 3.3.2. Correlations

IGF-1 levels were positively correlated with CPCs mainly for the same day or the subsequent days. Specifically, IGF-1 levels on day 1 were correlated with HSCs and eEPCs of the same day (rho = 0.512/*p* < 0.005 and rho = 0.412/*p* < 0.05, respectively). Likewise, IGF-1 levels on day 18 were correlated with HSCs and eEPCs of that day (rho = 0.525/*p* < 0.01 and rho = 0.524/*p* < 0.01, respectively). IGF-1 levels on day 1 were also correlated with lEPCs of day 18 (rho = 0.613/*p* < 0.005), where IGF-1 levels on day 18 were correlated with lEPCs of day 45 (rho = 0.477/*p* = 0.05). Finally, only borderline correlations of IGF-1 on day 18 were observed with VSELs on days 18 and 45. A low Apgar score at 1’ minute was correlated with low IGF-1 on day 9 (rho = 0.459/*p* < 0.05).

It seems that increased IGF-1 correlates positively with all progenitor cell lines on the same or the subsequent days, while perinatal stress and brain injury correlate with low IGF-1 levels in the following days. It is possible that IGF-1 serves as the intermediate connection between brain injury and low CPCs, i.e., one of the reasons that CPCs are low in patients.

### 3.4. Stromal Cell-Derived Factor-1

#### 3.4.1. Kinetics

SDF-1 levels were borderline higher in severe encephalopathy on day 9 and significantly higher in mild encephalopathy on day 18, compared to controls (Table 4 and Figure 2c). To further investigate the chemotactic axis SDF-1/CD184^+^, we also calculated the CD184^+^ cells/CD184^−^ cells ratio, which represents an increase or decrease in the CXCR4 receptor (of the SDF-1 ligand) on the cell surface. Compared to controls, the CD184 receptor was found to be increased in the subgroup of moderate encephalopathy on days 9, 18, and 45 (although statistical significance was not reached) and decreased in the subgroup of severe encephalopathy on days 3 and 9 (*p* < 0.05 for both, after Bonferroni correction) (Figure 2d). Additionally, CD184^+^ expression on day 9 was significantly higher in severe encephalopathy compared to moderate encephalopathy (after Bonferroni correction).

#### 3.4.2. Correlations

SDF-1 levels on day 18 were borderline correlated with lEPCs on day 45 (rho = 0.451/*p* = 0.08). SDF-1 levels were decreased on days 3 and 9 in neonates with late sepsis (*p* < 0.05 for both). Low pH after birth and increased hospitalization time were correlated with low SDF-1 on day 45 (rho = 0.689/*p* < 0.05 and rho = 0.547/*p* = 0.08, respectively).

### 3.5. Hematopoietic Stem Cells

#### 3.5.1. Kinetics

**Figure 3:**
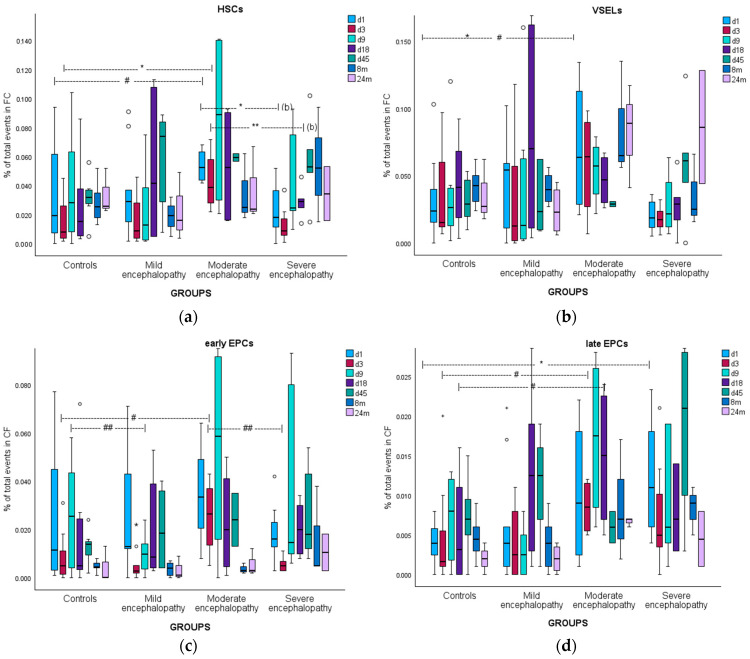
CPC kinetics in full-term controls and in neonates with encephalopathy: (**a**) HSCs; (**b**) VSELs; (**c**) early EPCs; and (**d**) late EPCs. FC: flow cytometry. * *p* < 0.05; ** *p* < 0.01; # trend (0.05 < *p* < 0.08); and ## trend not statistically significant after Bonferroni correction.

Patients showed significantly increased circulating HSCs only on day 45 of life compared to controls. However, when the patient group was divided into discrete subgroups of moderate and severe encephalopathy, it became clear that the kinetics of the two groups were indeed different: neonates with moderate encephalopathy had increased HSCs continuously from day 1 to day 45, while neonates with severe encephalopathy were unable to mobilize HSCs from day 1 to day 18. The latter reached increased levels compared to controls only on day 45 (*p* = 0.08), similar to levels found in neonates having moderate encephalopathy (Figure 3a). It is clear that in cases of severe brain damage, there is a complete inability to mobilize HSCs early on, at least until the 19th–45th day of life, when the endogenous repair system (regarding HSCs) “recovers” and reaches levels similar to those in cases with less severe tissue injury, which have more favorable prognosis.

#### 3.5.2. Correlations

A borderline decrease in HSCs on days 1, 3, and 18 of life was observed after caesarian section or multiple gestation (note that no correlation between caesarian section and multiple gestation was found in the current study). Moderate acidosis (pH between 7 and 7.2) during the first hours of life was correlated with significantly increased HSCs on day 9. Additionally, the four neonates who did not undergo therapeutic hypothermia had marginally lower HSCs on day 18 of life (*p* = 0.07). Although this is based on only four patients, it might be an indication that therapeutic hypothermia favors HSCs’ mobilization in the days after temperature recovery and thus supports endogenous rehabilitation mechanisms, which is one possible mechanism through which therapeutic hypothermia works. The same observation was also seen in EPCs.

### 3.6. Very Small Embryonic-like Stem Cells

#### 3.6.1. Kinetics

Circulating VSELs did not differ among groups, except at the age of 2 years of life, when they were significantly increased in patients. However, when a subanalysis was conducted, a pattern similar to that of HSCs was observed: there was a trend for increased mobilization of VSELs after moderate brain injury and an inability to mobilize VSELs in severe cases (Figure 3b). Mobilization at 24 months of age in neonates with encephalopathy might indicate chronic and persistent rehabilitation effort.

#### 3.6.2. Correlations

Borderline lower levels of VSELs were observed in neonates with late sepsis on days 9, 18, and 45 of life (0.08 < *p* < 0.084 for all). This suggests that inflammation and stress depress VSEL mobilization.

### 3.7. Early Endothelial Progenitor Cells

#### 3.7.1. Kinetics

eEPCs’ kinetics did not differ significantly among the groups. However, after a subanalysis, it became clear that EPCs were mobilized only in cases of moderate encephalopathy, mainly on days 1 to 9, with much less mobilization in the severe or mild encephalopathy groups (i.e., the same pattern as in HSCs) (Figure 3c).

#### 3.7.2. Correlations

Neonates that underwent therapeutic hypothermia showed borderline lower levels of eEPCs on day 3 (i.e., during the last day of hypothermia) (*p* = 0.08), and significantly higher circulating eEPCs on day 18 (*p* < 0.05), indicating pathways through which therapeutic hypothermia might benefit tissue regeneration.

### 3.8. Late Endothelial Progenitor Cells

#### 3.8.1. Kinetics

Patients showed increased mobilization of lEPCs at all time points, with statistical significance on days 1 and 3. After a subanalysis, it became clear that lEPCs were increased in both moderate and severe encephalopathy in the same manner (Figure 3d).

#### 3.8.2. Correlations

A low pH after birth and a low Apgar score at 1’ minute were marginally correlated with higher levels of lEPCs on days 1 and 9. Additionally, lower levels of lEPCs on days 3 and 9 of life were correlated with subsequent sepsis (*p* < 0.05 and *p* = 0.06, respectively). Specifically, lEPC levels below 0.0005‰ of total events in flow cytometry provided 100% sensitivity and 87.1% specificity regarding an upcoming sepsis (AUC: 0.935, 95% CI: 0.842–1, *p* < 0.05).

### 3.9. Brain Injury Biomarkers

Serum S100B levels were increased only in the subgroup of neonates with severe encephalopathy, while NSE levels were significantly correlated with the grading of encephalopathy, providing excellent prognostic ability, as shown in detail earlier [97].

#### 3.9.1. Correlations with Chemoattractants

S100B levels were positively correlated with EPO and SDF-1 levels for the same or the subsequent days and were adversely correlated with IGF-1 levels. Similarly, NSE levels were more consistently positively correlated with EPO and SDF-1 levels for the same or subsequent days and adversely correlated with IGF-1 levels (Appendix A). It appears that brain injury events themselves upregulate chemoattractants EPO and SDF-1, while the trophic factor IGF-1, vital for body and brain development, is inhibited.

#### 3.9.2. Correlations with CPCs

S100B on day 1 (when S100B levels were highest) was consistently negatively correlated with VSELs at any time point during the neonatal period: S100B(d1) with VSELs(d1) (rho = −0.527/*p* < 0.001), VSELs(d3) (rho = −0.401/*p* < 0.05), VSELs(d9) (rho = −0.371/*p* < 0.05), VSELs(d18) (rho = −0.435/*p* < 0.05), and VSELs(d45) (rho = −0.629/*p* < 0.001). Additionally, S100B levels on day 18 were adversely correlated with HSCs on day 18 (rho = −0.518/*p* < 0.001) and with lEPCs on days 18 (rho = −0.597/*p* < 0.05) and 45 (rho = −0.584/*p* = 0.077).

NSE levels only showed positive correlation with EPCs: NSE(d3) with eEPCs(d3) ((rho = 0.319/*p* = 0.066) and eEPCs(d18) (rho = 0.436/*p* < 0.05); NSE(d9) with eEPCs(d18) (rho = 0.338/*p* = 0.058); NSE(d18) with eEPCs(d18) (rho = 0.597/*p* < 0.001); NSE(d3) with lEPCs(d3) (rho = 0.466/*p* < 0.01) and lEPCs(d18) (rho = 0.367/*p* = 0.08). This difference between S100B and NSE correlations is of great interest and is discussed below in detail.

### 3.10. MRI Imaging

Brain MRI was performed after the 1st week of life in 9 out of the 10 neonates with moderate/severe encephalopathy who survived. Seven neonates had a mean total brain injury score of 4 and a mean basal ganglia injury score of 2.57, two neonates had a parasagittal injury score of 5 (severe), while two neonates had a total brain injury score of 0 (no detectable injury) (Appendix B). Brain MRI injury scores (basal ganglia, parasagittal injury, or total injury score) were steadily negatively correlated with all scales and subscales of the Bayley III developmental score (−0.624 < rho < −0.528, and *p* < 0.05 for all), as expected: the more severe the brain injury, the lower the score in the developmental test, affecting all scales and subscales.

### 3.11. Long-Term Neurodevelopmental Outcome

Eight infants with moderate/severe encephalopathy, six infants with mild encephalopathy, and five controls were assessed between 24 and 30 months of age with the Bayley III developmental test. Infants with moderate/severe encephalopathy had lower scores in all domains compared to controls and infants with mild encephalopathy (as described earlier [97]). Surprisingly, infants with mild encephalopathy achieved higher scores than controls in almost all domains (except the gross motor subscale), including the cognitive scale and in the fine motor subscale (*p* < 0.05 for both). It is possible that even a mild perinatal event (that eventually did not affect the brain) acted as a “wake-up call” for the families and led to more careful follow-up and daily positive interventions in those infants, resulting in higher developmental scores at the age of 2 years of life.

Circulating HSCs measured at the 8th month of age and eEPCs measured after the 24th month of age were steadily negatively correlated with almost all scales and subscales of the Bayley III test, depicting the long-term increased CPCs in patients (as described above), indicative of a long-term effort for tissue regeneration.

## 4. Discussion

The clinical challenges associated with neonatal encephalopathy demand innovative approaches that can go beyond the current therapeutic modalities. Progenitor cell therapy offers a promising strategy to address the underlying cellular damage in NE. The regenerative properties of HSCs, VSELs, eEPCs, and lEPCs provide multiple avenues for promoting tissue repair, reducing inflammation, and improving neurological function. However, several challenges remain. First, the optimal timing, dosage, and delivery methods for these progenitor cells need to be established to maximize their therapeutic potential. Additionally, further research is required to fully understand the mechanisms through which these cells exert their effects in the neonatal brain, including their ability to cross the blood–brain barrier, interact with resident neural cells, and integrate into damaged tissues. Despite these challenges, the promise of progenitor cell therapy in neonatal encephalopathy is compelling. A plethora of preclinical studies have shown the efficacy of progenitor cells in tissue repair [9,13,25,26,27]. Similarly, clinical studies on adults were very promising [43,52]. The first clinical trials in neonates have shown the short-term safety and indications of efficacy of these treatments [20,21,22]. Several insufficiently reported trials in infants and children are ongoing around the world, hoping to promote neuronal regeneration and avoid disability. Given the urgency of improving outcomes for neonates with encephalopathy, advancing the understanding and application of progenitor cell therapies could revolutionize the management of this condition.

To our knowledge, this is the first study on full-term neonates affected by neonatal encephalopathy that simultaneously investigates brain injury biomarkers, several chemoattractants, and CPCs at multiple time points in the acute, subacute, and chronic phases of brain injury, providing important insights into the interplay between those factors. Beyond brain injury biomarkers, brain injury was well illustrated using an MRI scoring system, short-term neurological condition, and long-term neurodevelopmental scores, acting as a strong base for correlating CPCs and chemoattractants with the brain injury event. Our results indicate that specific patterns of CPC mobilization, in conjunction with the levels of EPO, IGF-1, and SDF-1, are strongly associated with the severity of encephalopathy and may offer valuable information regarding pathophysiological pathways and outcome.

Specifically, regarding HSC kinetics, we observed steady HSC levels during the first weeks of life in healthy neonates, in agreement with previous reports [53]. Li et al. observed a rapid decrease in CD34+ cells in the first 6 h of life [106], but our sample on day 1 was obtained after the 12th hour of life. Interestingly, a clear distinct pattern between neonates with moderate and severe encephalopathy was shown. While neonates with moderate encephalopathy showed continuous mobilization of HSCs from day 1 to day 45, neonates with severe encephalopathy were unable to mobilize HSCs effectively until later in the study period (day 45), despite having levels similar to the moderate encephalopathy group at that time. This suggests a delayed endogenous repair mechanism in severe encephalopathy, potentially reflecting a more impaired capacity for the early mobilization of HSCs due to severe brain injury (hypothesis B). In preterm neonates, higher HSC levels were linked to better survival in conditions like respiratory distress syndrome [107], although the results are variable for conditions like bronchopulmonary dysplasia [53,108]. HSCs have also been found to be increased in periventricular leukomalacia of premature neonates [109], but observations for intraventricular hemorrhage are inconclusive [53,66,110].

As with HSCs, a similar pattern of increased mobilization of eEPCs and VSELs following moderate encephalopathy and reduced mobilization in severe cases was evident (hypothesis B). Even when not reaching statistical significance at many time points, repeated teleological meaningful patterns in a pilot study have to be highlighted for future research. The levels of VSELs increased in neonates with encephalopathy at 24 months of age, indicating a possible long-term effort for tissue rehabilitation. eEPCs primarily mobilized in neonates with moderate encephalopathy during the first 9 days of life, while those with severe or mild encephalopathy showed reduced mobilization. These findings are consistent with prior studies that indicate impaired stem cell responses in the context of severe brain injury in preterm neonates [110]. Finally, lEPCs showed increased mobilization across all groups, particularly on days 1 and 3, but also after 8 and 24 months, indicating a wider timeframe of regeneration effort and possibly for therapeutic intervention. In addition, both moderate and severe encephalopathy groups exhibited similar mobilization patterns for lEPCs, suggesting that their response may be less impacted by the severity of brain injury. This could be due to a different chemotactic axis and/or a different role of lEPCs, e.g., neo-angiogenesis in the penumbra. In previous studies on premature neonates, low EPCs were found in “stress” conditions, such as gestational diabetes, preeclampsia, chorioamnionitis, sepsis, necrotizing enterocolitis, and bronchopulmonary dysplasia [54,60,66,68,70,111,112], but these observations were not consistent among studies [113].

The impaired capacity of CPCs to be mobilized might be due to damage in the microenvironment of the neurons, i.e., astroglia and endothelial cells, from which the chemoattractants are released in cases of neuronal death [38,91], leading to chemoattractant axis failure and inability of CPC mobilization. This theory is interestingly supported by the correlation between S100B, NSE, and CPCs in our study: we observed an adverse correlation between S100B and CPCs and a positive correlation between NSE and CPCs. Keeping in mind that (a) neurons are more vulnerable to hypoxia than astrocytes [1] and that (b) NSE is a marker of neuronal injury while S100B is a marker of astrocyte injury, the underlying pathophysiology is depicted: in cases of less severe hypoxia, only neurons are affected (i.e., increased NSE), leaving its microenvironment intact (including astrocytes), from which the chemoattractants are released, conveying the “help” message to the periphery and mobilizing CPCs. In that case, increased NSE levels correlate with increased CPC levels for the following days (hypothesis E), but in cases that the hypoxic event is severe enough to also affect the (less vulnerable) astrocytes (i.e., increased S100B), the chemoattractants cannot be released from the latter and no CPCs’ mobilization will follow (i.e., increased S100B will correlate with low CPCs). An illustration of brain injury biomarkers’ kinetics is supportive of this theory [97]. Delineating the underlying pathophysiology in detail in a personalized setting for every single neonate with encephalopathy, i.e., which tissues are most affected and when, will provide knowledge for setting up a personalized regenerative therapeutic plan in the future.

Additionally, severe brain damage affects the suprachiasmatic nucleus, sympathetic nervous system, and release of catecholamines, which are known to activate the chemoattactant axis SDF-1/CD184 and CPC mobilization (via G-CSF) [38,114], but they also act as chemoattractants for CPCs in a complex brain–bone–blood triad with paracrine actions in bone marrow’s progenitor cells [115]. Consequently, the more severe the neuronal injury is, the more it impairs the mobilization of peripheral progenitor cells and the capability of the regeneration of the damaged tissue. Finally, it is fairly obvious that multiorgan failure, metabolism dysfunction, and a general critical condition in cases of severe encephalopathy may prevent mobilization of CPCs per se. It seems that there is a critical point of brain injury before which endogenous regenerative mechanisms are working effectively and there are no severe long-term consequences, such as in moderate encephalopathy. After this point, endogenous repair mechanisms shut down and neurodevelopmental problems arise. Delineating those mechanisms and their critical point is the key to enhancing the defective endogenous repair system before it collapses, and it must occur at the right time and in the right way. Administering peripherally exogenous progenitor cells hoping to home CNS and reducing tissue injury will probably fail if the chemoattractant axis is malfunctioning or if neo-angiogenesis is not performed in the penumbra via EPCs (in order for the new cells arriving at the penumbra to be viable). In conclusion, it is very likely that if endogenous CPCs are not mobilized toward the damaged tissue, neither would the peripherally administered exogenous progenitor cells, and consequently, any attempt for stem cell transplantation will fail. This is probably the reason that so many attempts worldwide for rapidly administering stem cells have not shown any positive results. Delineating pathophysiology will provide clues for future targeted therapeutic intervention.

Moreover, in this study, certain clinical factors were found to influence CPC mobilization (hypothesis G). First, acidosis at birth correlated with increased eEPCs at later time points, while HSCs were increased (particularly on day 9), interestingly, only in cases of moderate acidosis. To be noted, in the current study, pH at birth did not differ significantly between subgroups of moderate and severe encephalopathy, indicating acidosis as a possible independent stress factor that influences CPCs’ mobilization. Similarly, other studies have shown increased umbilical cord progenitor cells after stress during delivery (prolonged second stage of labor and dystocia) [116,117]. These are indications that moderate stress triggers intrinsic defensive mechanisms. Additionally, late sepsis was associated with decreased VSEL levels at several time points, suggesting that inflammation and severe stress may suppress the mobilization of these cells, inhibiting their rehabilitation capacities. This observation was also clearly shown earlier in preterm neonates with encephalopathy, where any kind of inflammation (chorioamnionitis, late sepsis, or necrotic enterocolitis) were constantly correlated with depressed CPCs (HSCs, VSELs, and EPCs) for the whole first one and a half month of life [110]. Finally, lower levels of lEPCs at days 3 and 9 were predictive of subsequent sepsis, highlighting the potential utility of lEPCs as a biomarker for infection in neonates.

It should be highlighted that a significant obstacle that inhibits a comparison between studies is the inconsistency in the literature regarding cell surface characterization of each cell line, specifically, HSCs and, more importantly, EPCs, making it difficult to extract definite conclusions [56,118,119].

In the current study, the observed trend of marginally lower HSCs in neonates who did not undergo therapeutic hypothermia suggests that therapeutic hypothermia may promote HSC mobilization in the days following temperature recovery, supporting its potential role in facilitating endogenous neurorepair. It is possible that the reduced tissue injury in the neurons and their microenvironment after therapeutic hypoxia (compared to neonates without such treatment) favors an intact chemoattractant axis and CPC mobilization in the following days, in line with the abovementioned hypothesis. Similarly, neonates who underwent therapeutic hypothermia showed a transient reduction in eEPCs on day 3, the final day of hypothermia, when brain function and general metabolism are reduced [120] (as explained in detail earlier), confirming the abovementioned hypothesis. However, higher levels of eEPCs by day 18 reinforce the hypothesis that hypothermia may aid in the recovery of the mobilization capacity of progenitor cells post-treatment. It is obvious that we cannot make conclusive reports with only four infants that did not undergo therapeutic hypothermia, but this observation is remarkable. To be noted, hypothermia is primarily an endogenous reaction in animals and humans after brain injury, named with the Greek name “anapyrexia” [30,31,32]. Therapeutic hypothermia (the only evidence-based effective therapeutic approach in neonatal encephalopathy that is a standard treatment in neonatal intensive care units worldwide) is indeed the imitation and enhancement of nature’s anapyrexia model.

To our knowledge, this is the first study on full-term neonates with encephalopathy focusing on EPO and CPCs. An interesting finding is the elevated levels of EPO in neonates with moderate and severe encephalopathy, particularly in the subgroup with severe encephalopathy (hypothesis A). In the latter subgroup, EPO correlated with VSEL and EPC mobilization, while such a correlation was not observed in other groups (hypothesis D). This increase in EPO, a well-known response to hypoxia, suggests that EPO may play a role in the mobilization of these cells following neonatal severe brain injury and, consequently, in the regeneration effort of the damaged tissue and its vasculature. These observations are in full accordance with previous studies [79] and with our earlier study on preterms with encephalopathy, where EPO was increased in the brain injury group compared to controls while also being correlated with CPCs exclusively in the encephalopathy group [110]. The observation that CPCs are mobilized by EPO only when severe brain injury has taken place, as well as the direct positive correlation of EPO levels with S100B and NSE levels, suggest that (in addition to hypoxia) brain injury itself might be an independent factor that triggers EPO release, as Juul et al. have also concluded [79]. It seems that both necrotic brain tissue and its surrounding hypoxic area (penumbra) lead to EPO release and subsequent EPC mobilization, indicating an effort for neo-angiogenesis, possibly in the penumbra. Making the penumbra area viable is crucial for the remaining cells and for other progenitor cells that will mobilize from the opposite hemisphere [6,13] and the periphery [14,16] as well as for targeted exogenous progenitor cell administration in the future. An increase in EPO in metabolic acidosis has also been reported earlier [60].

In contrast, IGF-1 levels showed a more complex pattern, with slightly lower levels in neonates with moderate and severe encephalopathy but gradual increases over time in all groups (hypothesis A), a pattern that was also observed earlier in preterms with encephalopathy [110]. In accordance with the published literature, IGF-1 levels were found to be decreased earlier in neonatal asphyxia when measured in the umbilical blood [121], on days 1–3 and 10 of life and at the end of the first month of life [122,123]. In a previous study, the size of brain structures and neurodevelopmental progress at two years of age were associated with higher IGF-1 levels in the first weeks after birth [124,125]. Considering the properties of this trophic factor, its correlation with all CPC lines suggests a potential role for IGF-1 in supporting progenitor cell survival and mobilization, as well as tissue repair, in the neonatal period (hypothesis D). The correlation between low Apgar scores and low IGF-1 levels at day 9 further supports the hypothesis that early systemic stress may hinder the neuroprotective effects of IGF-1 (hypothesis G), an observation that was extensively seen in preterms [110]. Additionally, its adverse correlation with S100B and NSE (hypothesis C) highlights that a significant neurotrophic factor [82,124] that mobilizes many CPCs is decreased in severe brain injury, indicating a reason for inadequate regeneration and a potential new therapeutic target for the future.

SDF-1, another key chemotactic factor involved in progenitor cell mobilization, showed variable kinetics across the groups (hypothesis A): borderline lower levels in neonates with encephalopathy immediately after the hypoxic event (on day 1) and slightly higher levels on the subsequent days (days 9 and 18). It seems that the devastating event initially inhibits this chemoattractant, but in the following days, the regeneration mechanisms rebound: damaged brain tissue triggers SDF-1 release, as shown by a correlation between early brain injury markers and later SDF-1 levels (hypothesis C). This is consistent with studies on adults [52,94,95], where increased SDF-1 is observed after tissue injury, but the related pattern in neonates is far more complicated [35,54,110], making the correlation between SDF-1 and CPCs difficult to interpret [53,70]. It is interesting that in preclinical models of brain injury, SDF-1 levels were elevated for much longer in adult animals [92,93] in contrast to models of neonatal brain injury, indicating a much shorter therapeutic window in neonates [7]. Notably, in our study, a decrease in SDF-1 was observed in neonates with sepsis, which underscores the role of inflammation in modulating progenitor cell dynamics (hypothesis G). In accordance, chorioamnionitis (an intrauterine inflammation) was reported to correlate with lower subsequent SDF-1 levels in preterm encephalopathy [35,110]. This point is indeed interesting because it delineates the pathways in which inflammation of any origin may hinder brain injury regeneration. Notably, sepsis was correlated with lower VSELs and lEPCs, as has been described above. In the related literature, it seems that serum SDF-1 levels are often difficult to explain, maybe because SDF-1 is released from both the bone marrow (aiming for CPC homing) and the injured tissue [91,115,126]. Consequently, although SDF-1 is a well described factor in preclinical studies, studies on humans were often unable to confirm these relationships; a steady correlation with CPCs was not able to be established in our study as well. In order to overcome this obstacle, we also studied SDF-1 receptor (CD184/CXCR4) expression. It is known that HIF-1a (which is released from the endothelium near the area of tissue damage [38,91]) upregulates SDF-1 and CXCR4 simultaneously, leading to CPCs’ recruitment in a chemotactic way. The CD184^+^/CD184^−^ ratio was correlated with SDF-1 levels in the current study, confirming a parallel relationship. Specifically, CD184^+^/CD184^−^ ratio analysis provided a more nuanced understanding of how SDF-1 signaling through the CXCR4 receptor is differentially regulated in neonates with moderate encephalopathy compared to those with severe encephalopathy. CD184 (CXCR4) receptor was upregulated in moderate brain injury and downregulated in the severe type, highlighting that CPCs’ mobilization through the SDF-1/CXCR4 axis is indeed intense in moderate encephalopathy (the 8-fold increase in CD184^+^ cells on day 9 observed in this group is noteworthy) and malfunctions in severe brain injury. In the latter, failure to upregulate CD184 receptor on the cell surface leads to failure in CPCs’ mobilization, maybe explaining in part the subsequent low tissue repair and long-term neurological consequences.

The main limitation of this study was its single-center design and the relatively small sample size with a long-term follow-up period, which may have led to type II statistical error and limited the ability to perform multivariate analysis. Where multivariate analysis was not possible and the statistical analysis indicated the presence of a significant confounding factor, the confirmation of observed correlations after the exclusion of neonates with these parameters was used to establish the results. Nevertheless, the absence of a correlation with several important perinatal characteristics (such as gestational age, birth weight, or type of delivery) also decreases the number of possible confounders. Factors affecting biochemical pathways in critically ill patients in the intensive care unit are numerous and difficult to control as there are numerous complications and various medications are administered. Ideal “sterile” conditions can only be created in a laboratory setting. Thus, in clinical research, only large-scale multicenter studies would have the statistical power to explore all the parameters. However, the main goal of this pilot study was to detect unknown associations and pose questions for further research, rather than reach definitive conclusions. For this reason, it was considered valuable to even report findings at the threshold of statistical significance when repeated teleological meaningful patterns appeared in order to highlight significant points for future research.

## 5. Conclusions

In conclusion, the dynamic interplay between circulating progenitor cells, chemoattractants, and brain injury biomarkers offers new perspectives on the pathophysiology of neonatal encephalopathy and its long-term implications. Even though this is a single-center study, our findings suggest that the early mobilization of CPCs, as reflected in the levels of HSCs, VSELs, and EPCs, may play a vital role in a successful recovery after neonatal encephalopathy, while delays or dysfunction in this process, particularly in severe encephalopathy, may contribute to failure of endogenous regenerative mechanisms and subsequent adverse neurodevelopmental outcomes. Further studies are needed to refine these biomarkers and their potential use in clinical practice for early prognosis and targeted individualized therapeutic interventions in neonates at risk for long-term sequelae.

## Figures and Tables

**Table 1:** Neonatal characteristics among groups.

Groups	Controls (Group 1)	Mild NE (Group 2)	Mode Rate/Severe NE (Group 3)	Comparisons between Groups (*p*)
n	12	11	13	
Gestational age (weeks) [Median (range)]	38 (4)	39 (4)	38 (4)	
Birth weight (gr) [Mean (SD)]	2962 (333)	3341 (445)	3098 (680)	1 vs. 2 (<0.05)
Male gender [n (%)]	5 (41.6%)	9 (81.8%)	8 (61.5%)	
Cesarean section [n (%)]	6 (50%)	5 (45.5%)	6 (46.2%)	
Multiple gestation [n (%)]	2 (16.7%)	0	0	
Apgar score 1 [Median (range)]	8 (1)	5 (7)	1 (3)	1 vs. 2 (<0.005) 1 vs. 3 (<0.005) 2 vs. 3 (<0.01)
Apgar score 5 [Median (range)]	9 (1)	8 (5)	5 (3)	1 vs. 2 (<0.005) 1 vs. 3 (<0.005) 2 vs. 3 (<0.05)
pH (first hours of life) [Mean (SD)]	7.36 (0.1)	7.3 (0.07)	6.9 (0.1)	1 vs. 3 (<0.01) 2 vs. 3 (<0.005)
SBE (mmol/l, first hours of life) [Mean (SD)]	−6.82 (3.14)	−8.47 (4.58)	−17.81 (4.96)	1 vs. 3 (<0.005) 2 vs. 3 (<0.005)
Sepsis [n (%)]	0	1 (9.1%)	1 (7.7%)	
Death [n]	0	0	3	

NE: neonatal encephalopathy, SD: standard deviation, and SBE: standard base excess.

**Table 2:** EPO levels (mIU/mL) in neonates with encephalopathy and in controls (mean/SD).

	GROUPS			Comparisons (*p*)
Time	Controls (Group 1)	Mild NE (Group 2)	Patients (Group 3)	
d1	17.03 (27.20)	133.06 (137.25)	148.24 (284.58) [3a: 33.74 (37.1), 3b: 230.03 (358.92)]	1–2 (0.062) 1–3 (<0.05) 1–3b (<0.05)
d3	3.3 (2.38)	3.47 (2.52)	308.78 (354.1) [3a: 45.26 (65.13), 3b: 440.54 (368.36)]	1–3 (<0.005) 1–3b (<0.005) *
d9	6.23 (4.96)	3.66 (2.03)	94.51 (248.11) [3a: 4.68 (5.44), 3b: 139.42 (302.02]	
d18	7.23 (6.6)	5.57 (2.49)	6.32 (7.89) [3a: 6.88 (10.9), 3b: 5.87 (5.87)]	
d45	13,71 (5,49)	10.02 (5.17)	13.22 (8.02) [3a: 18.85 (12.52), 3b: 10.4 (4.83)]	
8 m	13.35 (4.31)	6.7 (3.05)	12.14 (3.69) [3a: 12.14 (3.69), 3b: -]	

3a: moderate encephalopathy group; 3b: severe encephalopathy group; d: day; and m: month. * Statistically significant after Bonferroni correction (*p* < 0.05).

**Table 3:** IGF-1 levels (ng/mL) in NE (mean/SD).

	GROUPS			Comparisons (*p*)
Time	Controls (Group 1)	Mild NE (Group 2)	Patients (Group 3)	
d1	27.82 (9.58)	24.37 (2.54)	24.02 (3.52) [3a: 25.43 (5.46), 3b: <23]	
d3	30.04 (8.84)	34.26 (13.87)	<23 [3a: <23, 3b: <23]	1–3 (0.072)
d9	75.17 (49.45)	41.49 (25.74)	36.04 (25.53) [3a: 34.55 (10.14), 3b: 37.04 (33.3)]	1–3 (0.08)
d18	51.7 (23.99)	54.83 (26.8)	52.59 (24.88) [3a: 40.32 (8.75), 3b: 62.4 (30.16)]	
d45	74.11 (16.09)	62.65 (24.33)	60.23 (27.46) [3a: 64.95 (30.33), 3b: 58.34 (29.75)]	

**Table 4:** SDF-1 levels (pg/mL) among groups (mean/SD).

	GROUPS			Comparisons (*p*)
Time	Controls (Group 1)	Mild NE (Group 2)	Patients (Group 3)	
d1	1139.1 (605.6)	865.2 (483.2)	794.5 (430.3) [3a: 430.6 (235.6), 3b:1085.7 (300.2)]	1–3a (0.065)
d3	1499.6 (359.1)	1496 (443.4)	1485.8 (581.6) [3a: 1447 (291.4), 3b: 1500.4 (676.9)]	
d9	1328.9 (283.8)	1519.4 (476.5)	1713.7 (495.6) [3a: 1304 (71.7), 3b: 1918.5 (489.8)]	1–3b (0.064)
d18	1244.9 (262.7)	1578.1 (216.4)	1276.9 (199.7) [3a: 1264.2(132.5), 3b: 1286.4(260.4)]	1–2 (<0.05)
d45	1485.9 (258)	1362.8 (335)	1264.6 (270.9) [3a: 1076.6 (52.2), 3b: 1339.8 (291)]	1–3a (0.08)
8m	1777.5 (230.4)	1719.12(265)	1613.71 (216.26) [3a: 1540.1 (194.1), 3b: 1834.5]	

**Figure A1:**
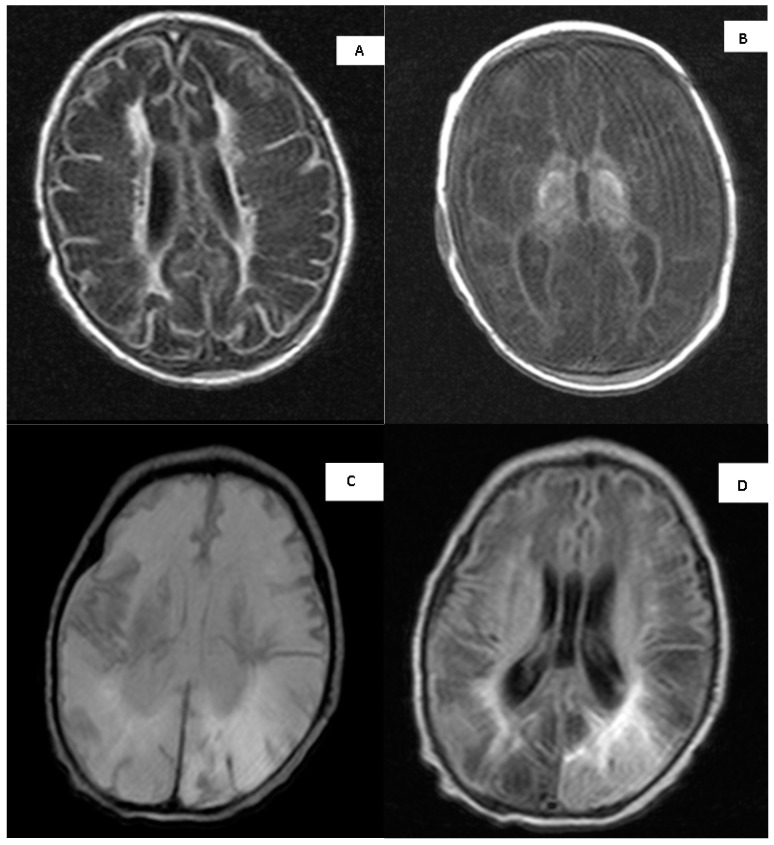
(**A**,**B**): Brain MRI images of patient no. 5 (showing hemorrhagic elements and complete obliteration of the grey matter–white matter boundary/liquefaction of the brain parenchyma). (**C**,**D**): Brain MRI images of patient no. 6 (hemorrhagic and ischemic lesions in the basal ganglia and parasagittal cortex).

## Data Availability

The original data presented in this study are openly available at: https://freader.ekt.gr/eadd/index.php?doc=36587#p=1 (accessed on 13 January 2025), (pp. 75–128). The dataset is available upon request from the authors.

## Appendix A

Correlation of S100B with chemoattractants:

- S100B(d1) with EPO(d3) (rho = 0.312/*p* = 0.08)
- S100B(d1) with EPO(d9) (rho = 0.427/*p* = 0.06)
- S100B(d3) with EPO(d9) (rho = 0.506/*p* < 0.05)
- S100B(d9) with EPO(d45) (rho = 0.426/*p* = 0.078)
- S100B(d18) with EPO(d45) (rho = 0.617/*p* = 0.077)
- S100B(d1) with SDF-1(d9) (rho = 0.486/*p* < 0.05)
- S100B(d3) with SDF-1(d9) (rho = 0.567/*p* < 0.01)
- S100B(d9) with SDF-1(d18) (rho = 0.642/*p* < 0.005).
- S100B(d1) with IGF-1(d9) (rho = −0.472/*p* < 0.05).

Correlation of NSE with chemoattractants:

- NSE(d1) with EPO(d3) (rho = 0.554/*p* < 0.005)
- NSE(d3) with EPO(d3) (rho = 0.467/*p* < 0.01)
- NSE(d3) with SDF-1(d9) (rho = 0.486/*p* < 0.05)
- NSE(d9) with SDF-1(d9) (rho = 0.467/*p* < 0.05)
- NSE(d1) with IGF-1(d3) (rho = −0.387/*p* < 0.05)
- NSE(d3) with IGF-1(d3) (rho = −0.391/*p* < 0.05)
- NSE(d9) with IGF-1(d9) (rho = −0.457/*p* < 0.05)

## Appendix B

**Table A1:** Brain MRI scoring of the 9 patients.

Patient	Basal Ganglia Injury Score	Parasagittal Injury Score	Total Injury Score
1	0	0	0
2	2	0	2
3	1	0	1
4	3	0	3
5	4	5	9
6	3	5	8
7	2	0	2
8	0	0	0
9	3	0	3
